# Consistent microbial responses during the aerobic thaw of Alaskan permafrost soils

**DOI:** 10.3389/fmicb.2025.1654065

**Published:** 2025-11-10

**Authors:** Joy M. O’Brien, Nathan D. Blais, Hannah Holland-Moritz, Katherine L. Shek, Thomas A. Douglas, Robyn A. Barbato, Jessica Gilman Ernakovich

**Affiliations:** 1 Department of Molecular, Cellular, and Biomedical Sciences, University of New Hampshire, Durham, NH, United States; 2 Center of Soil Biogeochemistry and Microbial Ecology, University of New Hampshire, Durham, NH, United States; 3 Department of Natural Resources and the Environment, University of New Hampshire, Durham, NH, United States; 4 U.S. Army Cold Regions Research and Engineering Laboratory, Fort Wainwright, AK, United States; 5 U.S. Army Cold Regions Research and Engineering Laboratory, Hanover, NH, United States

**Keywords:** permafrost microbiology, permafrost thaw, soil microbes, 16S bacterial ribosomal RNA, carbon flux

## Abstract

Arctic systems are warming at four times the global average, causing permafrost—permanently frozen soil, ice, organic matter, and bedrock—to thaw. Permafrost thaw exposes previously unavailable soil carbon and nutrients to decomposition—a process mediated by microbes—which releases greenhouse gases such as carbon dioxide and methane into the atmosphere. While it is well established that thaw alters the composition and function of the permafrost microbiome, patterns revealing common responses to thaw across different permafrost soil types have not yet emerged. In this study, we address how permafrost thaw impacts microbiome diversity, alters species abundance, and contributes to carbon flux in the Arctic. We sampled peat-like, mineral, and organic-mineral permafrost from three locations in central and northern Alaska. We assessed their abiotic soil properties and microbiome characteristics before and after a 3-month laboratory microcosm incubation. Across all sites, prokaryotic biomass increased following thaw, measured as 16S rRNA gene copy number. This change in biomass was positively correlated with cumulative respiration, indicating an increase in microbial activity post-thaw. We evaluated the thaw response of microbial taxa across three sites, identifying taxa that significantly increased in abundance post-thaw. Common responders shared across all sites belonged to the families *Beijerinckiaceae*, *Burkholderiaceae*, *Clostridiaceae*, *Oxalobacteraceae*, *Pseudomonadaceae*, and *Sporichthyaceae*, indicating a common set of taxa that consistently respond to thaw regardless of site-specific conditions. Alpha diversity decreased with thaw across all sites, likely reflecting the increased dominance of specific thaw-responsive taxa that may be driving post-thaw biogeochemistry and increased respiration. Taken together, we deepen the understanding of different permafrost microbiomes and their response to thaw, which has implications for the permafrost–climate feedback and enables more accurate predictions of how Arctic ecosystem structure and function respond to change.

## 1 Introduction

High-latitude ecosystems are undergoing rapid environmental change due to increasing global temperatures. These ecosystems are warming approximately four times faster than the rest of the globe (Rantanen et al., 2022), leading to the degradation of sea ice, glaciers, and permafrost (IPCC, 2019). Permafrost is defined as frozen soil, ice, organic matter, and bedrock that remain at or below 0 °C for two or more consecutive years (Van Everdingen, 1998; Schuur et al., 2008). Permafrost harbors a diverse microbiome consisting of dead, dormant, and active microbes across bacterial, archaeal, and fungal lineages (Burkert et al., 2019; Hansen et al., 2007; Steven et al., 2006; Waldrop et al., 2025). Permafrost covers approximately 15% of the northern hemisphere (Obu, 2021) and contains approximately twice as much carbon (C) as there is in the atmosphere (Hugelius et al., 2014; Nadeem et al., 2024), making it a critical component of the global climate system. For some microbes, permafrost soil carbon (C) and nitrogen (N) are largely inaccessible and not easily metabolized due to the frozen conditions. However, when permafrost thaws, soil temperature and moisture rise, and the accessibility of carbon and other soil nutrients increases (Ernakovich et al., 2017; Schädel et al., 2016).

The release of C following thaw—comprising particulate organic matter (POC), dissolved organic carbon (DOC), and/or mineral-associated organic matter (MAOM)—can stimulate microbial activity and decomposition, leading to increased microbial respiration and biomass (Clein and Schimel, 1995; Elberling and Brandt, 2003; Ernakovich et al., 2017; Mackelprang et al., 2011; Schädel et al., 2016). As microbes break down organic matter in thawing permafrost, they produce carbon dioxide, methane, and nitrous oxide as byproducts of aerobic and anaerobic respiration (Ernakovich et al., 2017; Hopkins et al., 2013). These greenhouse gasses are released into the atmosphere and contribute to positive carbon–climate feedback, further accelerating global warming (Schädel et al., 2016; Schuur et al., 2015). Therefore, understanding how changes in microbial abundance, composition, and respiration occur is imperative to accurately predict the contributions of permafrost thaw to atmospheric carbon dioxide and to make further predictions about climate change in the Arctic (Graham et al., 2012).

Previous permafrost thaw microcosm experiments (e.g., controlled temperature incubations) have shown significant changes in the factors affecting the composition of the permafrost microbiome in a relatively short period (weeks to months) (Barbato et al., 2022; Doherty et al., 2020; Ernakovich et al., 2017; Mackelprang et al., 2011; Ricketts et al., 2020). For example, in an incubation of soils from a peatland-dominated permafrost site in northern Sweden, depth and temperature were significant drivers of community composition post-thaw, which was also strongly influenced by drift—a stochastic assembly process driven by random birth, death, and reproduction (Doherty et al., 2020; Zhou and Ning, 2017). In contrast, the corresponding active-layer soils were dominated by deterministic assembly, where non-random selection by abiotic and biotic conditions drives composition, implying a predictable post-thaw community composition (Doherty et al., 2020; Ernakovich et al., 2022). Understanding the consistency and predictability of thaw responses is important for inferring the functional capabilities of post-thaw communities.

Similarly, identifying which microorganisms consistently proliferate following thaw could help predict the post-thaw permafrost microbiome composition and function. Microbes that increase in abundance following thaw can be classified as “thaw responders”—a distinct set of taxa that respond similarly to permafrost thaw. Previous research has shown that permafrost microbes exhibit variations in temperature tolerance, and there is potential for the pre-frozen microbiome to respond favorably when conditions improve (Ernakovich et al., 2017, 2022; Waldrop et al., 2023). For instance, one permafrost incubation study identified the emergence of nitrogen-fixing methanogens post-thaw, along with an increase in members of the phyla Actinobacteria, Proteobacteria, Bacteroidetes, and Firmicutes (Mackelprang et al., 2011).

Another study observed a positive correlation between Alphaproteobacteria and the amount of mineralized carbon, along with heightened microbial activity in response to readily available nutrients. This study also reported an increase in the abundance of Betaproteobacteria, Gammaproteobacteria, and Bacteroidetes (Ricketts et al., 2020). However, whether there is a common set of microbes or functional groups that increase consistently with permafrost thaw is unknown. Therefore, seeking out the identity of “thaw responders” promises to enable an understanding not only of post-thaw community composition but also of post-thaw gas fluxes. While permafrost encompasses a diverse range of soils, from mineral to organic, which support varied microbiomes (Ping et al., 2015; Waldrop et al., 2023, 2025), identifying common responders is a promising way to advance knowledge across this diversity.

The goal of this study was to understand how permafrost thaw impacts the microbiome and its contribution to carbon dioxide flux, using microcosm experiments with samples from three sites in Alaska. We performed a 96-day thaw microcosm experiment with permafrost from three different sites (ranging from mineral to organic permafrost and varying in carbon content). Throughout the incubation, we measured microbial respiration and flushed to prevent anoxia; cumulative carbon dioxide is presented as the total accumulated over time. We determined bacterial abundance via quantitative polymerase chain reaction (qPCR) and community composition via amplicon sequencing of the 16S rRNA gene before and after thaw. We hypothesized that (1) bacterial abundance and respiration would increase following thaw across sites, and (2) a core group of thaw responders would consistently emerge and increase in absolute abundance across sites post-thaw.

## 2 Materials and methods

### 2.1 Site descriptions, sample collection, and processing

Permafrost samples were collected from three locations in Alaska and stored at several institutions before being shipped to the University of New Hampshire (Table 1). Two permafrost cores were collected per site. The first site is located above the U. S. Army Cold Regions Research and Engineering Laboratory (CRREL) Permafrost Tunnel (PT), 10 km north of Fairbanks, AK. This site represents syngenetic permafrost with high carbon and ice content, primarily of eolian origin, and has a typical active layer depth of ~60 cm (Douglas et al., 2021). It is located along a gently sloping upland area. Cores from above the tunnel were collected on October 4, 2020. The second site is the CRREL Farmers Loop (FL) Experimental Station in Fairbanks, AK, which represents a lowland syngenetic, ice-rich permafrost with a higher sand content than that of the tunnel site. The active layer at this site is typically ~80 cm deep (Douglas et al., 2020). Cores from the FL site were collected on May 26, 2018. The two Fairbanks-area sites represent discontinuous permafrost. The third location is the Barrow Experimental Observatory (BEO), located on an extremely low-gradient plane 5 km southeast of Utqiaġvik, AK. Permafrost at the BEO is continuous (up to 400 m thick) and comprises sand, silt, and peat layers, with typical active layer depths of ~ 45–50 cm and 50.8 cm at the time of collection (Douglas and Blum, 2019; Nelson et al., 2008; Thurston et al., 2025). Cores from the BEO were collected on September 26 and 28, 2018. All three sites contain ice-rich permafrost, which can be broadly described as soil particles cemented by pore ice, with massive ice wedges. This type of permafrost is carbon-rich and susceptible to the formation of large thermokarst features upon thaw due to its high ice content.

**Table 1:** Permafrost sample collection metadata.

Sample site in AK	Date of sampling	Coordinates	Description	Permafrost donor, Storage location prior to this study
Above CRREL Permafrost Tunnel (PT)	October 4, 2020	64.951147 N, 147.620516 W	Mineral permafrost (% C < 20)	Tom Douglas, CRREL
CRREL Farmers Loop (FL)	May 26, 2018	64.874775 N, 147.681356 W	Organic/peat-like permafrost (%C > 20)	Taylor Sullivan, Andy Parsekian, University of Wyoming
Barrow Experimental Observatory (BEO)	September 24, 26 2018	71.323 N, 156.6114 W	Organic and mineral mix/sandy and silty cryoturbate (%C > 20)	Robyn Barbato, CRREL

Mineral permafrost indicates an organic layer of <40 cm. Organic permafrost indicates an organic layer of >40 cm.

All permafrost cores were collected using a gas-powered 8 cm diameter SIPRE corer (Jon’s Machine Shop, Fairbanks, AK, United States). Cores were approximately 1 meter in length (typically in four ~25 cm core sections). To protect permafrost samples from contamination, all personnel wore coveralls (Tyvek, Wilmington, DE, United States) and nitrile gloves during sample collection. At all three sites, the seasonally thawed active layer soil was removed using shovels, and cores were collected to a depth of 1 meter from the active layer-permafrost boundary. Samples were kept frozen in the field and remained frozen during transport from Alaska to various institutions, including the University of New Hampshire (UNH; Durham, NH, United States). When not in transport, samples were stored at −20 °C. At UNH, permafrost samples were aseptically prepared for incubation in a freezer room (−7 °C). Sterile conditions were maintained by cleaning the room, surfaces, and tools with 70% ethanol and 10% bleach. Personnel wore coveralls and nitrile gloves. To remove potential contamination, a sharp stainless steel paint scraper sterilized with 70% ethanol was used to scrape off the outer 0.5 cm of each core. After scraping for sterilization, we sectioned off each core with a sterile PVC wire saw for homogenization. Scraped and sectioned cores, approximately 25 cm in length, were placed in Whirl-Pak bags and stored at −20 °C until further use.

The same section of each core (0–25 cm) was removed from its Whirl-Pak bag, placed into a sterile (autoclaved) canvas bag (approximately 35.5 cm long x 12.7 cm wide), and hit with a sterilized hammer until fully homogenized. Canvas bags were laundered and autoclaved between uses of each sample. We sampled four replicate subsamples (20–40 g) from each homogenized core for a total of 24 samples for incubation (3 sites x 2 cores per site x 4 subsamples per core = 24 samples). Additional subsamples were retained and stored at −20 °C as a pre-thaw comparison. Due to limited amounts of soil, we prioritized replication in pre-thaw microbial analyses over soil analyses, saving one pre-thaw subsample per core for soil analyses and four pre-thaw subsamples per core for microbial community analyses.

### 2.2 Permafrost incubation

**Figure 1:**
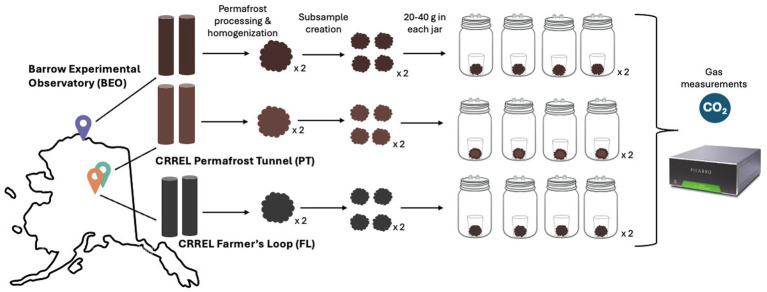
Permafrost incubation experimental design. Two permafrost cores were collected from three different sites in Alaska, United States. Each core was processed, homogenized, placed into jars, and incubated at 2 °C for 96 days with intermittent gas measurements (the notation x 2 indicates that the process was performed for each core).

The subsamples were incubated at 2 °C for 96 days to gently induce thaw (Figure 1). We chose an incubation temperature of 2 °C to replicate near-freezing temperatures typical of early thaw in Arctic regions. Each subsample was aseptically placed into a specimen cup and then into a mason jar fitted with a Luer lock to enable gas measurements throughout the incubation. To avoid water saturation, three holes and a glass fiber filter paper were placed in the bottom of the specimen cup, which was then placed on an inverted tin foil weigh boat inside the mason jar. This setup ensured that the incubation remained aerobic. Gas measurements were taken on days 0, 1, 2, 4, 6, 9, 12, 14, 19, 26, 30, 36, 44, 51, 58, 65, 74, 83, 92, and 96, and were analyzed for microbial respiration with a Picarro G2201-I cavity ring-down spectrometer (Picarro, Santa Clara, CA, United States). All materials used in the incubation were thoroughly sterilized prior to setup. To maintain CO_2_ concentrations below 2% and ensure aerobic conditions, the jar headspace was flushed with Ultra Zero air (Airgas, Dover, NH, United States) for 10 min after each gas measurement. After each measurement, the jars were randomly rearranged in the incubator to minimize potential positional effects. Respiration rate was calculated as μg CO_2_-C g^−1^ dry soil h^−1^. Dry soil weight was calculated using the gravimetric water content (GWC) of the samples, and cumulative respiration was expressed as μg CO_2_-C g^−1^ dry soil.


$$\begin{array}{l} \mathrm{\mu g}\;CO_{2}-Cg^{-1}\mathrm{dry}\;\text{soil}\;h^{-1}=\frac{CO_{2}\;\text{μmol}}{\mathrm{mol}\;\mathrm{air}}\\ {}*P*V*\frac{1}{R}*\frac{1}{T}*\frac{1}{g\;\mathrm{dry}\;\text{soil}}*\frac{12\;\mathrm{\mu g}\;C}{1\;\text{μmol}\;C}*\frac{1}{t} \end{array}$$

We calculated the respiration rate using the following parameters: *P* represents atmospheric pressure (atm), *V* is the volume of headspace (L), *R* is the ideal gas constant (L atm K_−1_ mol_−1_), *T* is the incubation temperature (K), and *t* is the incubation duration (hours) since the most recent flush.

### 2.3 Soil analyses

After 96 days of incubation, the thawed permafrost subsamples were destructively sampled for downstream analyses. Both pre-thaw and post-thaw soils were weighed for the following measurements: 0.25 g for DNA extraction and 10 g for GWC, pH, and electrical conductivity (EC). To determine GWC, 10 g of soil was placed in a drying oven at 105 °C for 48 h and then weighed (Gardner, 1965). The dried soil was transferred to a 50 mL Falcon tube for pH and EC analyses (Rayment and Higginson, 1992). For both analyses, 25 mL of Milli-Q water was added to each tube, and the tubes were placed on a tabletop shaker for 1 h at 115 rpm. EC was measured first using an Accumet AB30 Conductivity Meter equipped with an Accumet glass body conductivity cell (0.1 cm). pH was then measured with an Accumet Basic 15 pH Meter (Thermo Fisher Scientific, Waltham, MA, United States). Combustible total carbon and nitrogen were quantified in triplicate on dry soil samples using combustion analysis (Costech ESC 4010, Valencia, CA, United States) at 950 °C. Acetanilide was used for calibration alongside blanks, and an instrument check standard was run every 12 samples. The coefficient of determination (R^2^) was 0.999651 for nitrogen (entire range) and 0.999848 for carbon (entire range).

### 2.4 Microbial community analyses

DNA was extracted from the pre-thaw and post-thaw soil permafrost subsamples using the Qiagen PowerSoil Pro Kit (Qiagen, Hilden, Germany), following the manufacturer’s protocol. The DNA was then amplified in 12 μL reactions using PCR with the following primers and conditions: 515F with Nextera adaptors (5′TCGTCGGCAGCGTCAGATGTGTATAAGAGACAGGTGYCAGCMGCCGCGGTAA) and 926R with Nextera adaptors (5′GTCTCGTGGGCTCGGAGATGTGTATAAGAGACAGCCGYCAATTYMTTTRAGTTT) of the V4 regions of the 16S rRNA gene to identify bacterial and archaeal communities (Parada et al., 2016; Quince et al., 2011). The PCR reactions were set up as follows: 6 μL DreamTaq Hot Start Green (Thermo Fisher Scientific, Waltham, MA, United States), 2.6 μL of a sterile water solution containing 0.5 μg/μL bovine serum albumin (BSA) to reduce potential PCR inhibitors (Kreader, 1996), 0.7 μL of forward primer (5 μM), 0.7 μL of reverse primer (5 μM), and 2 μL of template DNA. The thermocycler conditions for 16S rRNA amplification were as follows: enzyme activation at 94 °C for 3 min, 35 cycles of denaturation at 94 °C for 45 s, annealing at 50 °C for 60 s, and extension at 72 °C for 90 s, and finally, a final extension at 72 °C for 10 min.

PCR products (including one blank) were then visualized via gel electrophoresis. During each round of DNA extraction, we included two empty tubes to serve as blanks. All samples were successfully amplified and sent to the University of New Hampshire Hubbard Genome Center in Durham, NH, United States, for library preparation via PCR, multiplexing, and sequencing on an Illumina NovaSeq 6000 (Illumina, San Diego, CA, United States) with 2 × 250 bp chemistry. A portion of the samples were resequenced to achieve an acceptable sequencing depth (>20,000 reads per sample). To control for run-to-run biases, several samples that were sequenced to sufficient depth on the first run were also resequenced on the second run.

Quantitative PCR (qPCR) was performed on all previously extracted DNA using a Bio-Rad CFX96 thermocycler (BioRad, Hercules, CA, United States). We aimed to quantify the 16S rRNA gene of the V4 region using the aforementioned primers (515F and 926R). DNA samples were normalized to 10 ng of DNA in 46 μL of molecular-grade water. Reaction volumes of 15 μL were set up as follows: 7.5 μL SsoAdvanced™ Universal SYBR® Green Supermix (BioRad, Hercules, CA, United States), 1.125 μL (10 μM) forward primer, 1.125 μL (10 μM) reverse primer, 3 μL template DNA, and 2.25 μL molecular grade water. The thermocycling protocol was carried out as follows: polymerase activation at 95 °C for 4 min, denaturation (40 cycles) at 95 °C for 30 s, annealing at 53 °C for 30 s, and extension at 72 °C for 60 s. Standard curves were composed of five 10-fold serial dilutions (0.836 to 0.00000836 ng/μL) of genomic DNA from *Escherichia coli* K-12 (Blattner et al., 1997), extracted from a pure culture (Carini et al., 2016). All samples and standards were run in triplicate. All reaction efficiencies were >104% and all *R*^2^ values were >0.986. Results were standardized to the original mass of soil and are reported as gene copies g^−1^ soil.

### 2.5 Sequencing and statistical analyses

The two sequencing runs were processed independently using the DADA2 pipeline for denoising and assembly into amplicon sequence variants (ASVs), with modifications to the error rate learning step for NovaSeq quality scores (Callahan et al., 2016; Teixeira et al., 2024). Briefly, primer sequences were removed with Cutadapt, and reads were filtered and trimmed for quality (quality plots determined the number of base pairs to trim). The resulting ASV tables were merged using the “mergeSequenceTables” function within DADA2. Chimeras were removed, and taxonomy was assigned with the Silva database v138. To account for sequence run-based differences in base calling, ASVs in the merged sequence table were aligned and then clustered at 99% sequence similarity using the “DECIPHER” and “speedyseq” R packages (Wright, 2016; McLaren, 2020). We reassigned taxonomy on the clustered ASVs using the “assignTaxonomy” function in DADA2 (Callahan et al., 2016).

The merged sequence table contained 19,617 ASVs. After clustering at 99% similarity, the final ASV table contained 17,073 ASVs across 52 samples (following the second merge of duplicate samples) and was used for all further statistical analyses in the “phyloseq” (McMurdie and Holmes, 2013) and “vegan” packages (Oksanen et al., 2022). All analyses were performed in R (version 4.2.0; R Core Team, 2021). Taxa unassigned at the phylum level were removed (1,025 removed), in addition to reads that were identified as mitochondria and chloroplasts (6,835), which were approximately 42.6% of the total reads. DNA extraction blanks and PCR blanks were sequenced alongside samples to assess contamination. Blanks yielded little to no reads; when ASVs were detected in blanks, these were removed from the dataset prior to downstream analyses (five sample blanks were removed from the dataset). We rarefied the samples to a depth of 5,000 (this depth ensured that the majority of the samples were retained). We removed the samples affected by lab error (FL1C4 REP 4 pre- and post-thaw samples).

Following sequencing, we employed a number of statistical analyses to evaluate the collected data. One-way analysis of variance (ANOVA; in the “vegan” package) was used to test for differences in the post-thaw soil abiotic factors by site, such as pH, EC, total carbon, and total nitrogen, followed by post-hoc analyses of between-group differences using Tukey’s HSD tests (in the “stats” and “multcompView” packages). For abiotic data that failed the assumptions of normality (Shapiro–Wilk test in the “stats” package) and homogeneity of variance (Levene’s test), we used the non-parametric Kruskal–Wallis test (in the “stats” package) for significance, followed by a Dunn test (in the “FSA” package) with *p*-values adjusted with Holm’s method for post-hoc comparisons. Correlations relating gene copy number to cumulative respiration were performed using the Spearman method via the “cor.test” function (in the “stats” package). All figures were made in ggplot2 (Wickham, 2016). Diversity metrics were calculated using the Shannon and Simpson indices, using the “vegan” package, followed by further analysis with the Kruskal–Wallis and Dunn tests. We measured community dissimilarity between prokaryotic communities using the Bray–Curtis dissimilarity measure and visualized it using principal coordinates analysis (PCoA) ordination plots.

Differences in community dispersion were assessed using the “betadisper” function (to assess homogeneity of dispersion), followed by a permutation test of multivariate homogeneity of groups using the “permutest” function (n = 999), and Tukey’s HSD test to determine pairwise comparisons across sites. PERMANOVA was performed using the “adonis2” function in the “vegan” package to test for differences in microbial communities by site and pre-post-thaw treatments. The relative abundance of taxa was scaled accordingly by the 16S rRNA gene counts from qPCR to estimate absolute microbial abundance (Jian et al., 2020). Response ratio analysis (Hedges et al., 1999) was used to identify the absolute abundance of ASVs that increase due to permafrost thaw (Fan et al., 2022). To verify the validity of our response ratio results, we performed the analysis using non-rarefied data, which was centered log-ratio (CLR) transformed using the “propr” package (Jin et al., 2023) prior to analysis, and found that our results are consistent with those obtained with the rarefied dataset, indicating that our findings are robust to the choice of rarefaction.

The ASV data frame used in the response ratio analysis was scaled up by 0.01 to eliminate zeros while maintaining the presence-absence relationship. Log response ratios (RR) were calculated as the natural log of post-thaw abundance divided by pre-thaw abundance. Standard errors were estimated for each RR, which were then used to calculate Z-scores and perform a two-tailed test to assess the statistical significance of each RR. *p*-values were corrected for false positives in multiple comparisons using the Benjamini & Hochberg false discovery method (Benjamini and Hochberg, 2000). Individual ASVs were considered significantly responsive if the FDR-adjusted *p*-value was <0.05. We assessed response ratios that showed a two-fold increase or more post-thaw.

## 3 Results

### 3.1 Pre-thaw and post-thaw abiotic analyses

Soil abiotic properties changed from pre-thaw to post-thaw and also varied within sites (Table 2). Pre-thaw pH across all sites was acidic, ranging from 4.16 to 4.91, while post-thaw pH ranged from 3.94 to 4.88. Post-thaw pH significantly decreased between BEO and PT (TukeyHSD *p*-value = 0.001), as well as between BEO and FL (TukeyHSD *p*-value = 0.001). Electrical conductivity (EC) ranged from 206.3 to 441.6 mS/cm before thaw. Following the thaw, EC values (228.7 to 478.5 mS/cm) were significantly lower at PT compared to BEO (ANOVA: *F* = 13.95, *p*-value = 0.0303, Tukey HSD *p* = 0.02). Pre-thaw total combustible carbon (% C) varied across sites, ranging from 1.8% at PT to 39.6% at FL. Pre-thaw total nitrogen (% N) also varied across sites in the same manner, with the lowest being 0.11% in PT samples and the highest at 2.15% in FL samples. Combustible C and N data were not taken on post-thaw soils due to a lack of samples. Prior to thaw, gravimetric water content (GWC) varied among sites, ranging from 61.6% in PT to 313.8% in FL. Following thaw, GWC decreased, ranging from 6.6% in PT to 263.9% in FL.

**Table 2:** Pre-thaw and post-thaw permafrost abiotic soil properties.

			Pre-thaw permafrost					Post-thaw permafrost			
Site	Core	pH*	EC (mS/cm)*	% C*	% N*	% C: N	% GWC *	pH	EC (mS/cm)	% GWC	Change in % GWC (pre-thaw to post-thaw)
PT	AT 3	4.84	350. 9	3.6	0.18	20	61.6	4.83 ± 0.03^b^	264.2 ± 57.7^b^	16.8 ± 7.4	−44.8 ± 7.4
PT	AT 4	4.91	245.3	1.8	0.11	16.4	91.9	4.88 ± 0.03^b^	228.7 ± 91.7^b^	6.6 ± 1.9	−85.3 ± 1.9
FL	FL 1	4.71	441.6	37.9	1.9	19.9	313.8	4.71 ± 0.02^b^	345.6 ± 37.5^ab^	263.9 ± 23.1	−49.9 ± 23.1
FL	FL 2	4.73	388.1	39.6	2.15	18.4	208.8	4.72 ± 0.05^b^	431.7 ± 25.1^ab^	146.6 ± 65.6	−62.2 ± 65.6
BEO	BEO 1	4.41	206.3	30.9	1.67	18.5	176.5	4.06 ± 0.05^a^	440.4 ± 84.5^a^	150.8 ± 41.5	−27.5 ± 41.5
BEO	BEO 2	4.16	328.6	21.8	1.07	20.4	116.9	3.94 ± 0.03^a^	478.5 ± 104^a^	129.3 ± 32.4	+12.4 ± 32.4

In the pre-thaw samples, “*” indicates that there were no subsamples for the respective measurements (one measurement per core due to sample limitations). Post-thaw samples were averaged across four replicates per core for pH, electrical conductivity (EC), and GWC measurements. The pH measurements were averaged for reporting using the formula −log_10[(ΣCi)/(n)], where C is the concentration of hydronium ions and n is the number of measurements (Boutilier and Shelton, 1980). Percent C and percent N measurements were not taken on post-thaw soils due to sample limitations. Water was freely drained from thawing permafrost before post-thaw GWC measurements (see Methods). Superscript letters after measurements indicate results from Tukey HSD tests, using a compact letter display, for statistically significant differences.

### 3.2 Pre-thaw and post-thaw bacterial abundance and cumulative respiration

**Figure 2:**
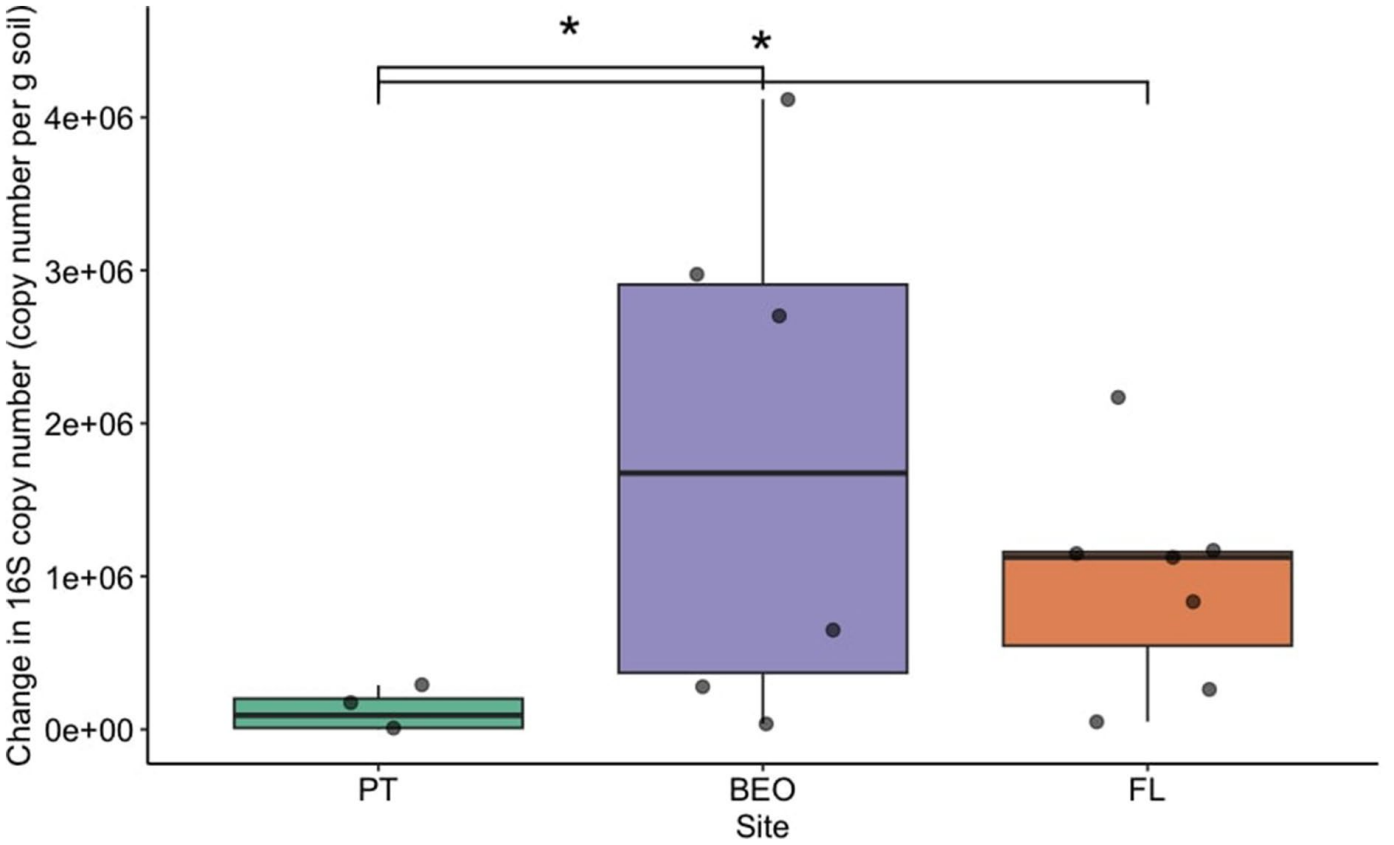
Bacterial abundance increased with thaw across all sites. Bacterial abundance was assessed via the change in 16S copy number before and after thaw, with significant differences between PT and BEO (Dunn adj. *p*-value = 0.02), as well as between PT and FL (Dunn adj. *p*-value = 0.03). Boxplots depict the median value as a solid line and the upper and lower quartiles as the range of the box. Whiskers indicate the extent of the data, and points represent raw data.

**Figure 3:**
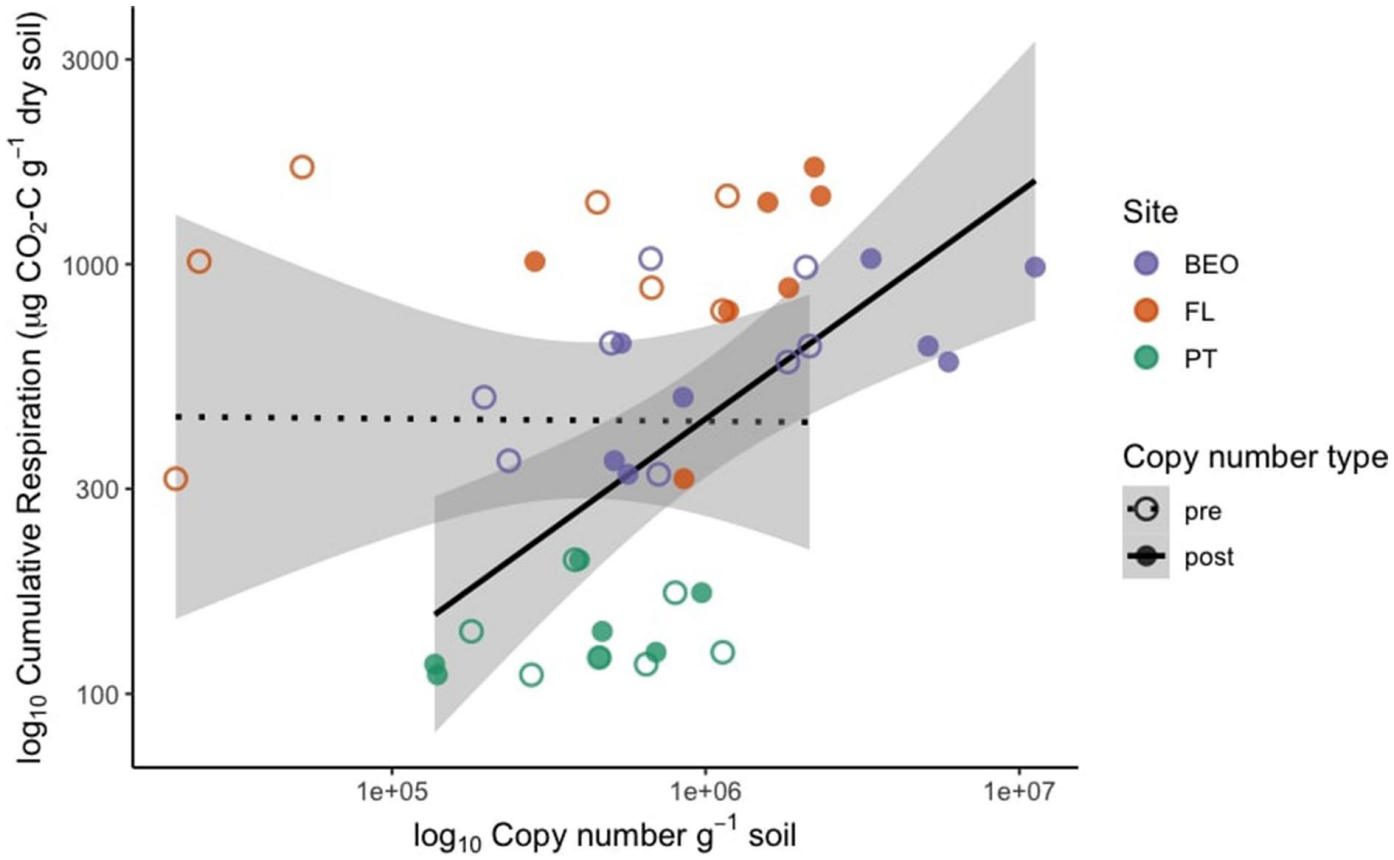
Cumulative respiration is positively correlated with post-thaw biomass, measured by 16S copy number. Pre-thaw Spearman correlation: rho = 0.11, *p* = 0.62; post-thaw Spearman correlation: rho = 0.66, *p* = 0.001.

Pre-thaw bacterial abundance was not significantly different across sites. However, the change in bacterial abundance with permafrost thaw was significantly different across sites (Figure 2) (Kruskal–Wallis *p*-value = 0.01). PT had the lowest change in biomass following thaw and was significantly lower than FL (Dunn adj. *p*-value = 0.03) and BEO (Dunn adj. *p*-value = 0.02), which had the most change in biomass with thaw, likely due to variability between cores. Cumulative respiration was also significantly different between sites (Supplementary Figure S1) (Kruskal–Wallis *p*-value = 0.0002). Cumulative respiration in PT samples was significantly lower than that of FL (Dunn *p*-value = 0.0001) and BEO (Dunn *p*-value = 0.007). We assessed the relationship between cumulative respiration and microbial abundance (gene copy number) (Figure 3). There was no significant correlation between cumulative respiration and pre-thaw copy number (Spearman’s rho = 0.11, *p*-value = 0.6). However, there was a significant positive correlation between cumulative respiration and post-thaw copy number (Spearman’s rho = 0.66, *p*-value = 0.001).

### 3.3 Post-thaw microbiomes across three sites

**Figure 4:**
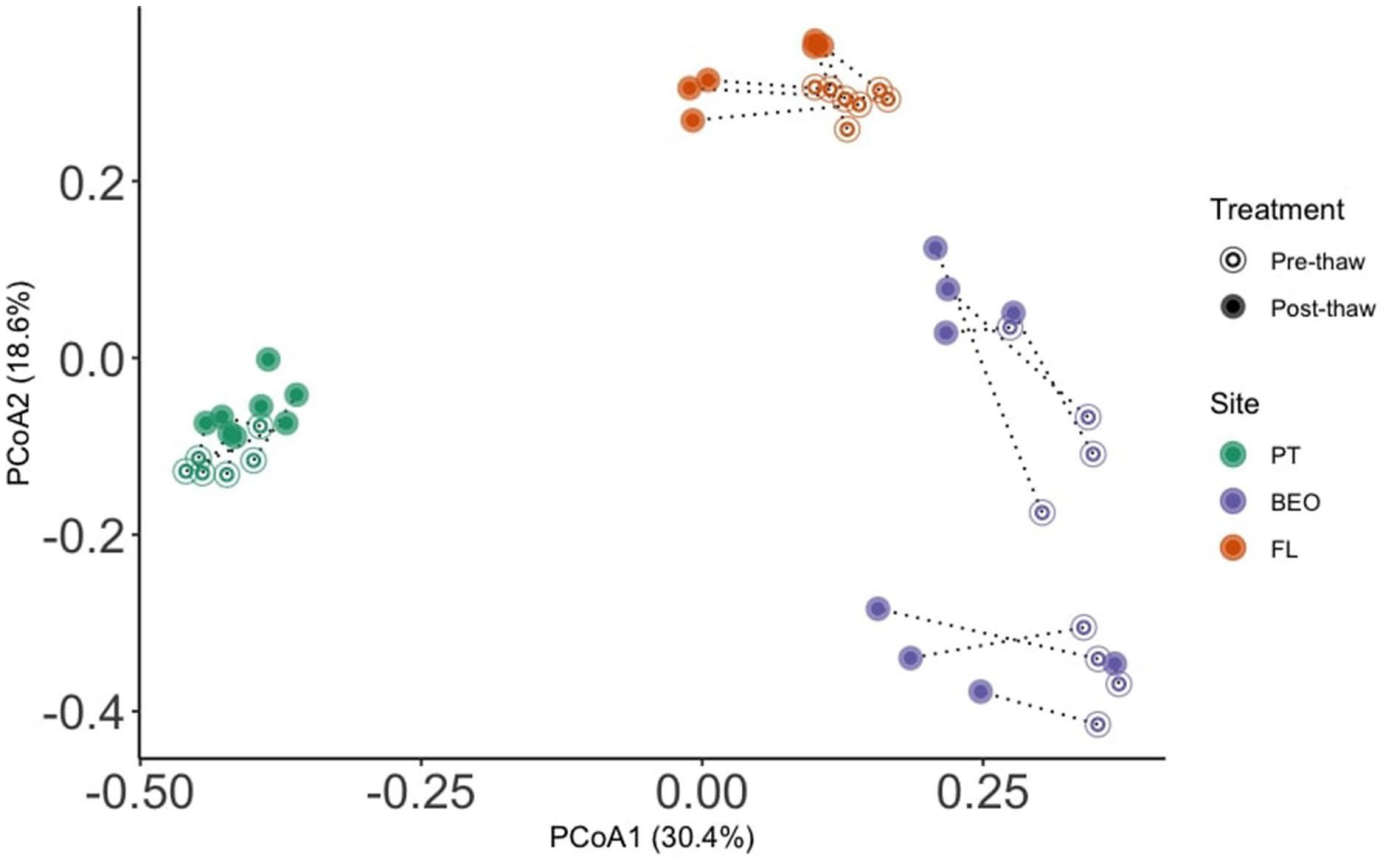
Microbial communities retain site-specific composition before and after permafrost thaw. PERMANOVA analysis revealed significant effects of the site (*R*^2^ = 0.44, *F* = 18.9, *p*-value = 0.001) and thaw (*R*^2^ = 0.08, *F* = 7.2, *p*-value = 0.001) on microbial community composition.

Microbial communities retain site-specific composition before and after permafrost thaw (Figure 4). Permutational analysis of variance (PERMANOVA) showed significant effects of the site (*R*^2^ = 0.44, *F* = 18.9, *p*-value = 0.001) and thaw (*R*^2^ = 0.08, *F* = 7.2, *p*-value = 0.001) on community composition. However, analysis of beta dispersion, followed by a permutational test for multivariate homogeneity of groups (“permutest”), indicated significant differences in dispersion among sites (*F* = 9.8, *p*-value = 0.001), suggesting that differences in community variability also contribute to the observed site effects. Pairwise comparisons showed greater dispersion at BEO compared to PT and FL (Tukey HSD adj. *p*-value = 0.001 for both).

**Figure 5:**
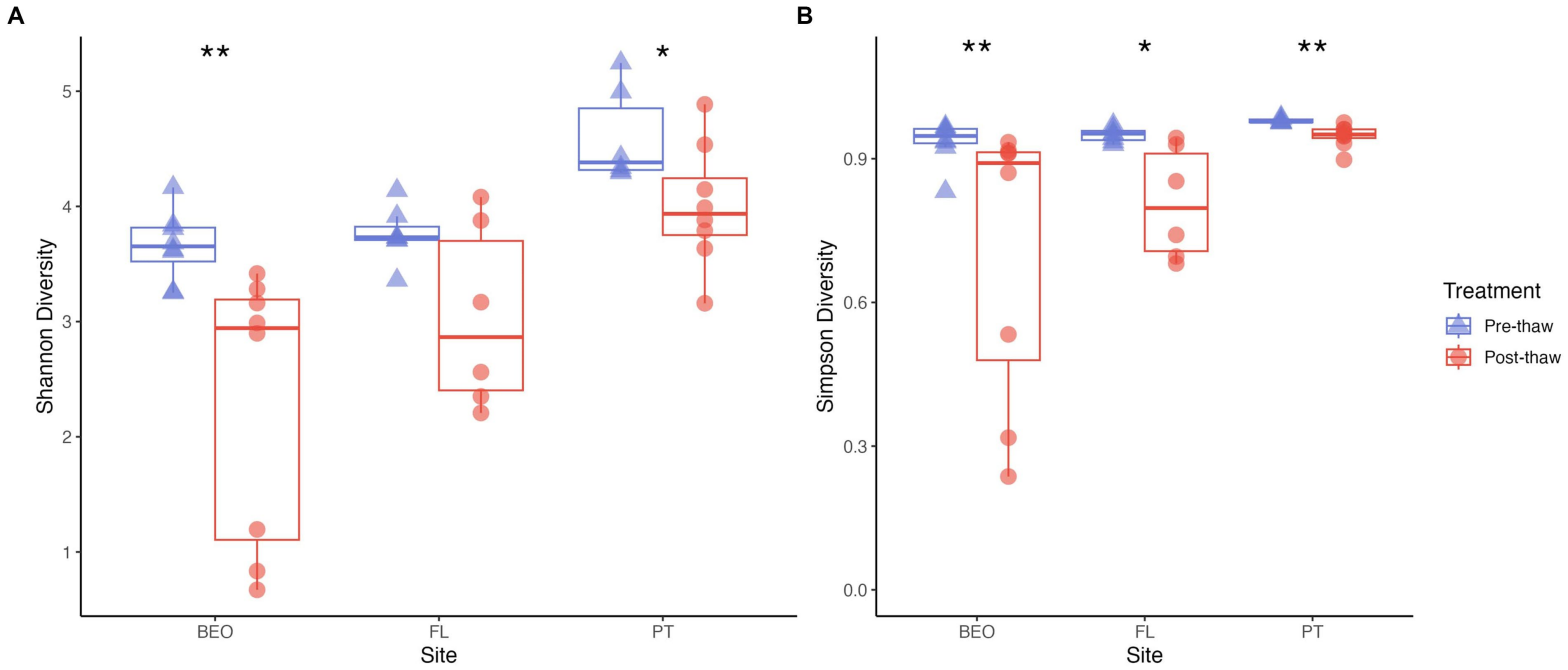
Alpha diversity decreases across all sites post-thaw. **(A)** Shannon and **(B)** Simpson diversity metrics for each site, pre-thaw and post-thaw. Asterisks indicate significant differences between groups.

Shannon diversity significantly differed between sites (Kruskal–Wallis *p*-value = 0.0001) and thaw treatment (Wilcoxon rank-sum test, W = 122, *p* = 0.007), with diversity decreasing with thaw (Figure 5A). Shannon values ranged from 3.25 to 5.25 in pre-thaw samples and from 0.67 to 4.89 in post-thaw samples. PT decreased by an average of 0.6 ± 0.25 post-thaw (ANOVA *F* = 5.171, *p*-value = 0.04), FL decreased by an average of 0.7 ± 0.33 post-thaw (Kruskal–Wallis *p*-value not significant), and BEO decreased by an average of 1.35 ± 0.25 post-thaw (Kruskal–Wallis *p*-value = 0.003). Similar trends to Shannon were observed with Simpson diversity, which was significantly different between sites (Kruskal–Wallis *p*-value = 0.0016) and thaw treatment (Wilcoxon rank-sum test, W = 81, *p*-value = 0.0001) (Figure 5B). Simpson values ranged from 0.83 to 0.99 in pre-thaw samples and from 0.24 to 0.97 in post-thaw samples. Further, Simpson diversity decreased with thaw as follows: PT decreased by an average of 0.032 ± 0.0085 (Kruskal–Wallis *p*-value = 0.002), FL decreased by an average of 0.14 ± 0.048 post-thaw (Kruskal–Wallis *p*-value = 0.01), and BEO decreased by an average of 0.23 ± 0.1 post-thaw (Kruskal–Wallis *p*-value = 0.006).

### 3.4 Analysis of thaw responders

**Figure 6:**
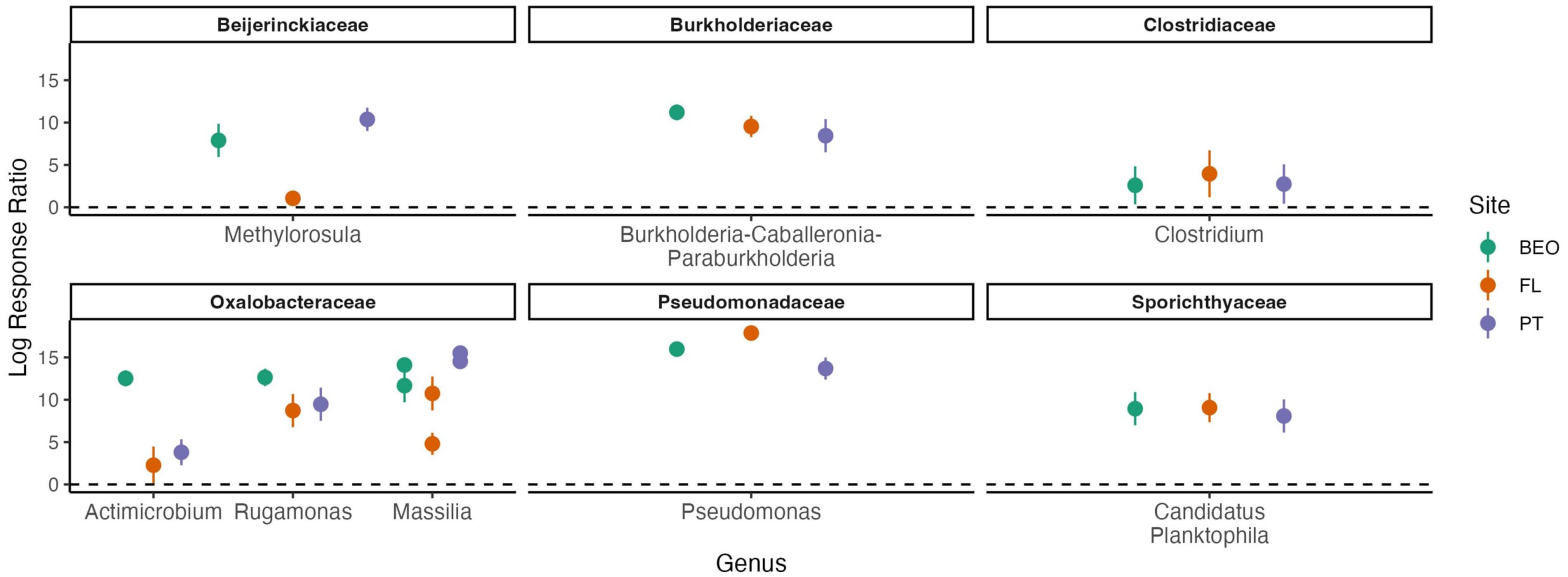
Positive thaw responder taxa, identified through response ratio analysis and shared across all sites, belong to the phyla Actinomycetota, Bacillota, and Pseudomonadota (all *p*-values < 0.001). We define thaw responders as bacterial taxa that increase in abundance post-thaw. Family and genus are listed in the figure titles and on the x-axis, respectively. Multiple points per genus indicate multiple species-level responses. Points represent the mean of replicates, and error bars show the confidence interval.

**Figure 7:**
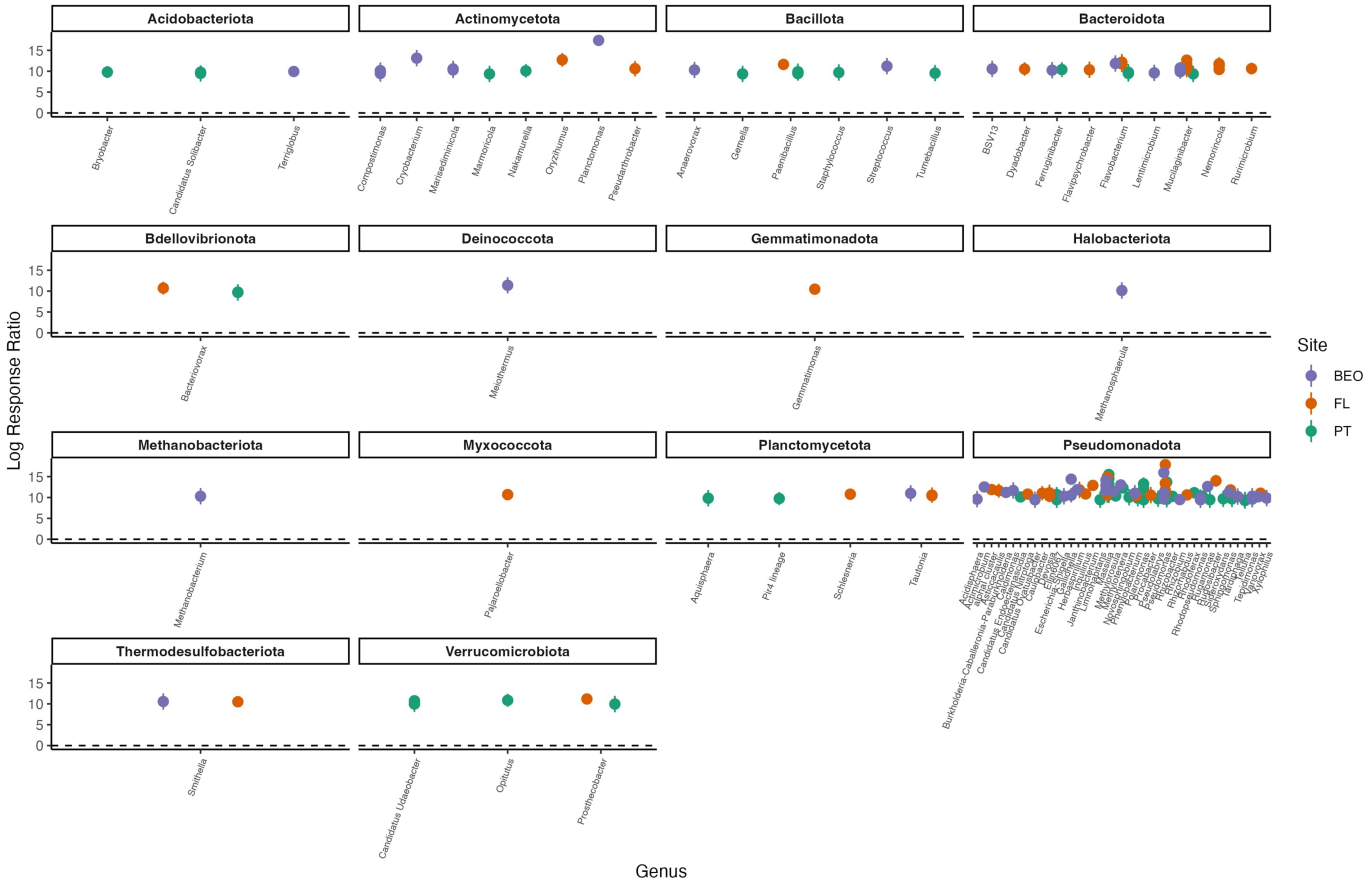
Positive thaw responder taxa, as determined by response ratio analysis, for PT, FL, and BEO sites. We define thaw responders as bacterial taxa that increased by at least twofold in abundance post-thaw. Phylum and genus are indicated in the figure titles and on the x-axis, respectively. Multiple points per genus indicate species-level responses. Points represent the means of replicates, and error bars show confidence intervals (all *p*-values < 0.001).

Response ratio analysis was used to assess common thaw responders across all sites (Figure 6). A positive response ratio value indicates that a particular ASV responded positively to thaw, meaning at least a two-fold increase in abundance relative to its abundance in the pre-thaw community (Fan et al., 2022). We found a total of eight common thaw responders from all sites at the ASV level and identified them at the genus level: *Methylorosula*, *Burkholderia-Caballeronia-Paraburkholderia*, *Clostridium*, *Actimicrobium*, *Rugamonas*, *Massilia*, *Pseudomonas*, and Candidatus *Planktophila* (all *p*-values < 0.05). Responders were distributed across three phyla—Actinomycetota, Bacillota, and Pseudomonadota—and together accounted for more than 50% of the post-thaw microbiomes at all sites (Supplementary Figure S4). We assessed positive thaw responders at each site individually and found 1,153 ASVs (490 from PT, 474 from FL, and 189 from BEO) from several other phyla that increased in abundance following thaw (all *p*-values < 0.05), including common thaw-responder phyla. In PT samples, these included Acidobacteriota, Bacteroidota, Bdellovibrionota, Chloroflexota, Methanobacteriota, Planctomycetota, Thermodesulfobacteriota, and Verrucomicrobiota. In FL samples, we observed increases in Acidobacteriota, Bacteroidota, Bdellovibrionota, Caldisericota, Gemmatimonadota, Myxococcota, Planctomycetota, Thermodesulfobacteriota, and Verrucomicrobiota. At BEO, thaw was associated with increases in Acidobacteriota, Bacteroidota, Caldisericota, Deinococcota, Halobacteriota, Methanobacteriota, Planctomycetota, Thermodesulfobacteriota, and Verrucomicrobiota (Figure 7).

## 4 Discussion

In this study, we investigated how permafrost thaw affects microbiome diversity, alters species abundance, and contributes to carbon flux across three distinct permafrost sites in Alaska through a microcosm incubation experiment. We found that (i) cumulative respiration and post-thaw microbial biomass were positively correlated, indicating that respiration has a strong relationship with new microbial growth; (ii) alpha diversity decreased following thaw across all sites; (iii) post-thaw microbiomes retain site-specific microbial community composition; and (iv) certain phyla—Actinomycetota, Bacillota, and Pseudomonadota—consistently increased in abundance following thaw across all sites. These findings corroborate previous permafrost incubation studies and highlight key taxa that respond to the favorable conditions of thaw regardless of site.

### 4.1 Cumulative respiration is positively correlated with an increase in bacterial abundance

Following the thaw, bacterial abundance increased across all sites (Figure 2), likely due to favorable conditions such as changes in temperature and soil abiotic properties like % C, % N, pH, and EC. These abiotic factors largely influence the structure and function of soil microbial communities (Fierer, 2017; Horner-Devine et al., 2007) and have a direct effect on cumulative microbial respiration. In particular, permafrost thaw makes soil C more bioavailable (Coolen et al., 2011; Ernakovich et al., 2015; Waldrop et al., 2010), which is vulnerable to microbial decomposition (Ernakovich et al., 2017; Schuur et al., 2015; Waldrop et al., 2010; Zimov et al., 2006). It is worth mentioning that other microbes (fungi, archaea, etc.) are present in permafrost and contribute to the cumulative respiration in addition to bacteria. We found that the increase in bacterial abundance was positively correlated with cumulative respiration (Figure 3), indicating that microbial growth and the larger biomass pool are driving permafrost C losses. Carbon content in permafrost is one of the key drivers of microbial respiration (Dutta et al., 2006; Schädel et al., 2016; Zimov et al., 2006), and because of that, as permafrost thaws, there are higher rates of microbial activity and increased respiration rates (Ernakovich et al., 2017; Mackelprang et al., 2011; Monteux et al., 2020; Schädel et al., 2016; Schuur et al., 2015). Other studies have indicated that the amount of organic substrate in permafrost has the potential to dictate microbial abundance and respiration (Müller et al., 2018; Stackhouse et al., 2015). Indeed, the amount of organic matter in the soil appears to be the dominant factor influencing bacterial abundance and respiration responses to thaw between our sites, as PT samples contained less C (~ 3%) and N (~ 0.15%) (on a per-mass basis) than FL (%C ~ 38%, %N ~ 2%) and BEO (%C ~ 26%, %N ~ 1.5%) for pre-thaw conditions (Supplementary Figures S2, S3). Taken together, our findings of the positive relationship between microbial abundance and cumulative respiration support other findings that the increase in soil microbial activity with thaw is due to the growth of microbial biomass (Coolen and Orsi, 2015; Ernakovich et al., 2017; Schädel et al., 2016; Schuur et al., 2015).

### 4.2 Post-thaw microbiomes are site-specific but contain common taxa with a consistent response to thaw

Following thaw, the post-thaw microbiomes across our three sites decreased in diversity, changed in composition, and increased in bacterial biomass, all of which have the potential to alter community function. This is consistent with previous studies that found that microbiome composition, functional gene abundance, and metabolic pathways change dramatically with permafrost thaw (Coolen and Orsi, 2015; Doherty et al., 2020; Mackelprang et al., 2011; Tuorto et al., 2014; Waldrop et al., 2025). Indeed, these changes are driven by assembly processes that shape the post-thaw microbiome, primarily dispersal, as thaw reduces dispersal limitations in natural settings (Doherty et al., 2025; Ernakovich et al., 2022). Our findings that alpha diversity decreased with thaw (Figure 5) are consistent with other studies (Thurston et al., 2025). FL and BEO had similar microbial diversity, as assessed with the Shannon and Simpson metrics. This may be due to similar soil properties (e.g., soil C and N).

Microbial diversity was significantly different between PT and both FL and BEO, which may further support the relationship between diversity and soil C and N. This decrease in diversity may reflect a combination of biological and abiotic processes following thaw. For example, thaw could increase the abundance of a few responsive taxa, reducing evenness and overall diversity. Consistent with this, microbial biomass increased, indicating a community composed of fewer but more abundant taxa, a pattern common in disturbance events (Ernakovich et al., 2022; Mackelprang et al., 2011; Seitz et al., 2021). Alternatively, relic DNA in intact permafrost could artificially inflate the apparent pre-thaw diversity; however, relic DNA has not been shown to shape the permafrost microbiome (Burkert et al., 2019). Previous work has shown that Shannon diversity in permafrost is related to soil pH, with lower diversity in more acidic conditions (Waldrop et al., 2023). Consistent with this, we found that our observed declines in diversity were associated with declines in pH levels. Taken together, the decline in diversity and the presence of thaw responders suggest that the rise in greenhouse gas production during thaw may result from the growth of particular taxa.

Understanding common microbial responses to thaw is essential for predicting the structure and function of post-thaw microbial communities. The power of our cross-site study lies in the fact that, despite site-specific differences in the pre- and post-thaw community compositions, we found common thaw responders (analyzed at the ASV level and identified at the genus level) that increased at least two-fold in abundance following thaw. We define thaw responders as bacterial taxa that increase in abundance post-thaw. An increase post-thaw is likely due to the favorable conditions that permafrost thaw presents (increase in labile carbon, moisture, and so on). There were a number of taxa from the phyla Actinomycetota, Bacillota, and Pseudomonadota that were common thaw responders across sites and increased in absolute abundance (Figure 6; Supplementary Figure S4). The phyla of the thaw responders observed in this study are consistent with other studies; Actinomycetota: (Chen et al., 2021; Mackelprang et al., 2011; Monteux et al., 2020; Scheel et al., 2023; Stackhouse et al., 2015), Bacillota (formerly known as Firmicutes): (Barbato et al., 2022; Chen et al., 2021; Coolen and Orsi, 2015; Monteux et al., 2020; Stackhouse et al., 2015; Yang et al., 2021), and Pseudomonadota (Kim et al., 2025). The responders belong to the genera *Methylorosula*, *Burkholderia-Caballeronia-Paraburkholderia*, *Clostridium*, *Actimicrobium*, *Rugamonas*, *Massilia*, *Pseudomonas*, and Candidatus *Planktophila*. Additionally, each site also has its own set of taxa that responded to thaw (Figure 7). Interestingly, the site-specific thaw responder results align with recent findings of the same phyla present in the atmosphere as bioaerosols above major permafrost thaw zones. These airborne microbes actively contribute to the Arctic aerobiome, which can lead to cloud formation, alter precipitation patterns, and affect radiative forcing (Nieto-Caballero et al., 2025). In this study, we focused on the roles and characteristics of these common thaw responders and what their responses may imply for ecosystems following thaw. These responders vary in their functional roles and characteristics, with some genera known for their pigment-producing properties, promotion of plant growth, and contributions to biogeochemical cycling.

#### 4.2.1 Actinomycetota

Candidatus *Planktophila, a* freshwater bacterioplankton genus, increased at all sites following thaw. This genus has no known isolates to date but has been studied in a stable mixed culture (Jezbera et al., 2009) and is described as an amino acid prototroph (Neuenschwander et al., 2018). This genus likely increases following thaw due to the increased water, which can promote the formation of thaw lakes where this genus is largely abundant (Bomberg et al., 2019; Vigneron et al., 2019). Indeed, all our permafrost samples were high in ice content and have the potential to contribute to large thermokarst features in natural settings.

#### 4.2.2 Bacillota

*Clostridium*, a genus within the phylum Bacillota, is a Gram-positive, spore-forming, anaerobic bacterium known for the potential pathogenicity of some of its species. Clostridium was likely dormant under the frozen conditions of permafrost, as it can enter dormancy through sporulation when conditions are unfavorable*. Clostridium* has been previously found to increase in abundance with thawing (Barbato et al., 2022; Chen et al., 2021; Coolen and Orsi, 2015; Yang et al., 2021), while other studies have noted a decrease in its abundance (Kim et al., 2025). The increase in permafrost thaw is likely due to spore germination, as they respond to favorable conditions for microbial growth and activity.

#### 4.2.3 Pseudomonadota

*Pseudomonas* is a genus of Gram-negative, aerobic bacteria found in a wide variety of environments such as soil, water, plant seeds, and animal hosts (Palleroni, 2008). A number of *Pseudomonas* strains have plant-growth-promoting properties. For instance, previous work has shown an increase in *Rhodoferax* sp. and *Gallionella* sp. following thaw, which largely influenced iron cycling in the post-thaw microbiome (Romanowicz et al., 2023). Additionally, recent work has revived potato plant pathogens from thawing permafrost, as these previously dormant bacteria increased with thaw (Kim et al., 2025).

*Methylorosula* is a Gram-negative, aerobic, motile bacterium within the family *Beijerinckiaceae*. It has one known species, *Methylorosula polaris*, originally isolated from acidic tundra wetland soils in Northern Russia. This genus is facultatively methylotrophic and not capable of fixing nitrogen (Berestovskaya et al., 2012). Instead, *Methylorosula* is known for its metabolic versatility because it can synthesize most carbon compounds, such as sugars, polysaccharides, ethanol, and amino acids, in addition to one-carbon compounds like methanol and methylamines (Berestovskaya et al., 2012). This genus is likely a thaw responder because these compounds are degraded during permafrost thaw via decomposition performed by other microbes. *Methylorosula*’s ability to process single-carbon compounds may aid in reducing methane emissions from permafrost, as it may limit substrates available to methanogens and regulate carbon fluxes.

*Burkholderia-Caballeronia-Paraburkholderia* (BCP) is one of the common thaw responders and has been previously noted to respond positively to thaw (Scheel et al., 2023). BCP refers to three closely related genera within the *Burkholderiaceae* family. The genus *Burkholderia* was recently subdivided into three clades: *Burkholderia* (clade I), which consists mostly of plant and animal pathogens; *Caballeronia* (clade II), which consists of environmental isolates; and *Parkaburkholderia* (clade III), to distinguish plant growth-promoting and root-associated bacteria (Madhaiyan et al., 2021; Sawana et al., 2014). Members of BCP are Gram-negative bacteria and are known for their plant growth-promoting properties as rhizobacteria (Madhaiyan et al., 2021). BCP contribute to organic matter decomposition, releasing CO_2,_ and some contribute to nitrogen cycling (*Paraburkholderia*). Some *Paraburkholderia* are capable of nitrogen fixation and can suppress phytopathogens, which may benefit plants in thawing permafrost ecosystems. The contribution of nitrogen fixation and plant growth promotion could enhance vegetation with permafrost thaw, potentially storing more carbon in the ground.

*Actimicrobium* is a Gram-negative, strictly aerobic, non-motile genus within the family *Oxalobacteraceae*. It has only one known isolate, *Actimicrobium antarcticum*, which was originally isolated from Antarctic coastal seawater (Kim et al., 2011). It was recently found to be in high abundance in Antarctic glacier boreholes (Cui et al., 2025). Similar to *Actimicrobium*, *Rugamonas* was also originally isolated from Antarctica, but from freshwater samples. *Rugamonas* is characterized as a genus of Gram-negative, aerobic bacteria. Recent research has shown that members of this genus produce violacein, a microbial antibiotic pigment (Choi et al., 2015; Sedláček et al., 2021). Similarly, *Massilia*, another positive thaw responder, also produces violacein (Sedláček et al., 2021).

## 5 Conclusion

As permafrost thaws, the microbiome’s composition, abundance, and diversity change. Collectively, these changes have the potential to accelerate global warming. Our results identify a positive relationship between cumulative respiration and post-thaw microbial biomass, decreased diversity following thaw, and common positive thaw responders, which likely contribute to the increase in respiration with thaw. These thaw responder taxa are known for producing antimicrobial pigments, promoting plant growth, and contributing to biogeochemical cycling. Their increased abundance can alter community dynamics and promote vegetation, which has implications for the carbon cycle. Future research is needed to further elucidate the functions of these positive thaw responders and the post-thaw microbiome, particularly their contributions to the permafrost carbon-climate feedback.

## Data Availability

The sequence data presented in this study can be found in the National Center for Biotechnology Information (NCBI): https://www.ncbi.nlm.nih.gov/bioproject/PRJNA1284569. The code, figures and additional data used for statistical analyses and figure generation is available via GitHub (https://github.com/joyobrien20/thaw.response).
