# Supplementary material for: Consistent microbial responses during the aerobic thaw of Alaskan permafrost soils
Source: Front Microbiol. 2025 Nov 10;16:1654065. doi: 10.3389/fmicb.2025.1654065 (PMC12643468; doi:10.3389/fmicb.2025.1654065)
Supplement: Supplementary file 1 [file Data_Sheet_1.docx]

Supplementary Material

# Supplementary Figures

**
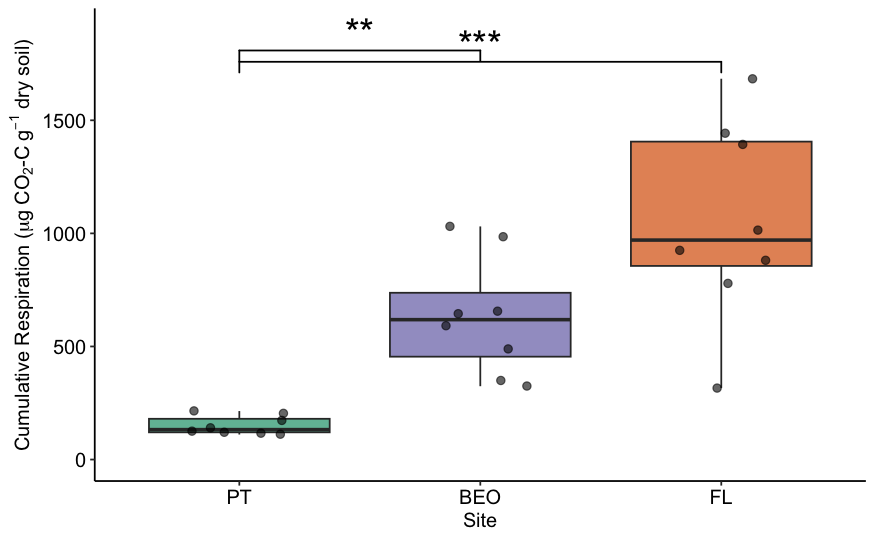
**

**Supplementary Figure 1.** Cumulative respiration during thaw varies among sites (Kruskal-Wallis p-value = 0.0002) with significant differences between CRREL and Farmer’s Loop (FL) (Dunn p-value = 0.0001) as well as CRREL and Utqiagvik (Dunn p-value = 0.007). Boxplots depict the mediavalue as a solid line and the upper and lower quartiles as the range of the box. Whiskers indicate the extent of the data and points represent raw data.

**
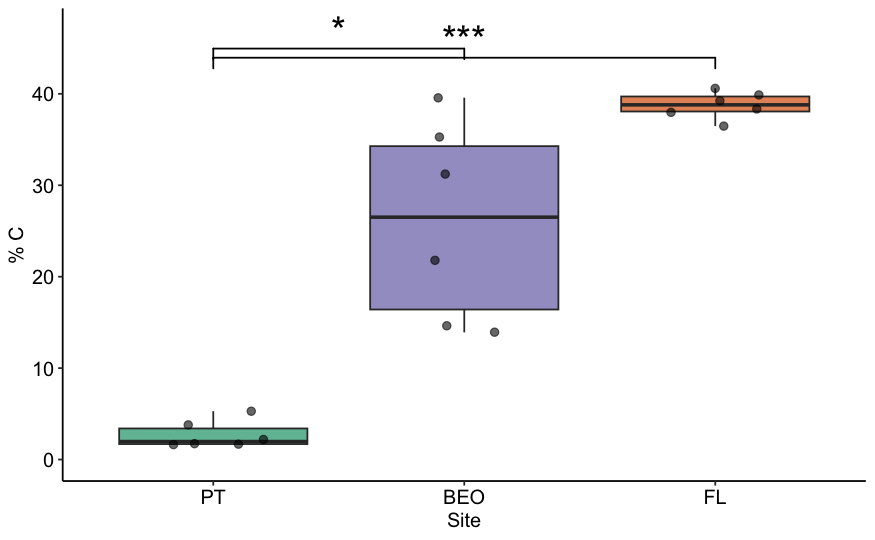
**

**Supplementary Figure 2.** Total carbon varies by site. Significant differences between CRREL and Utqiagvik (Dunn p-value = 0.03) and CRREL and Farmer’s Loop (FL) (Dunn p-value = 0.0002). Boxplots depict the median value as a solid line and the upper and lower quartiles as the range of the box. Whiskers indicate the extent of the data and points represent raw data.

**
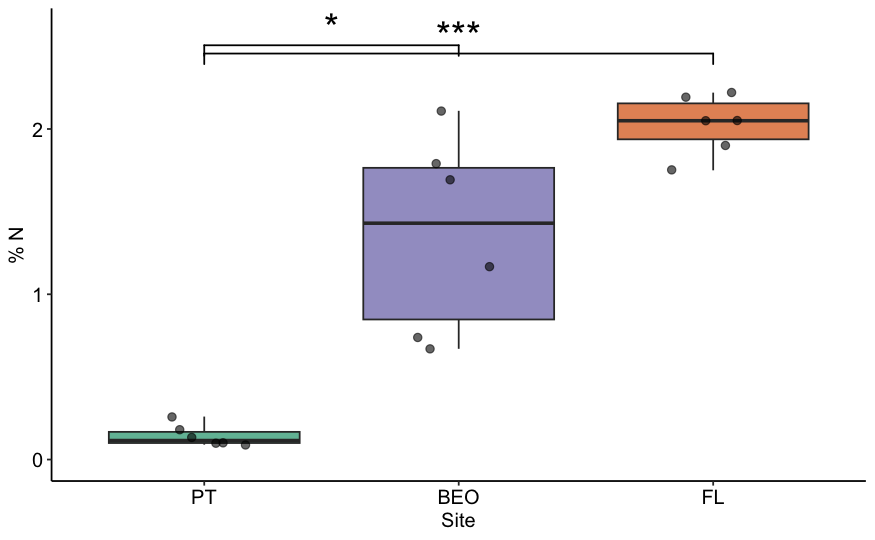
**

**Supplementary Figure 3**. Total nitrogen varies by site. Significant differences between CRREL and Utqiagvik (Dunn p-value = 0.02) and CRREL and Farmer’s Loop (FL) (Dunn p-value = 0.0002). Boxplots depict the median value as a solid line and the upper and lower quartiles as the range of the box. Whiskers indicate the extent of the data and points represent raw data.

**
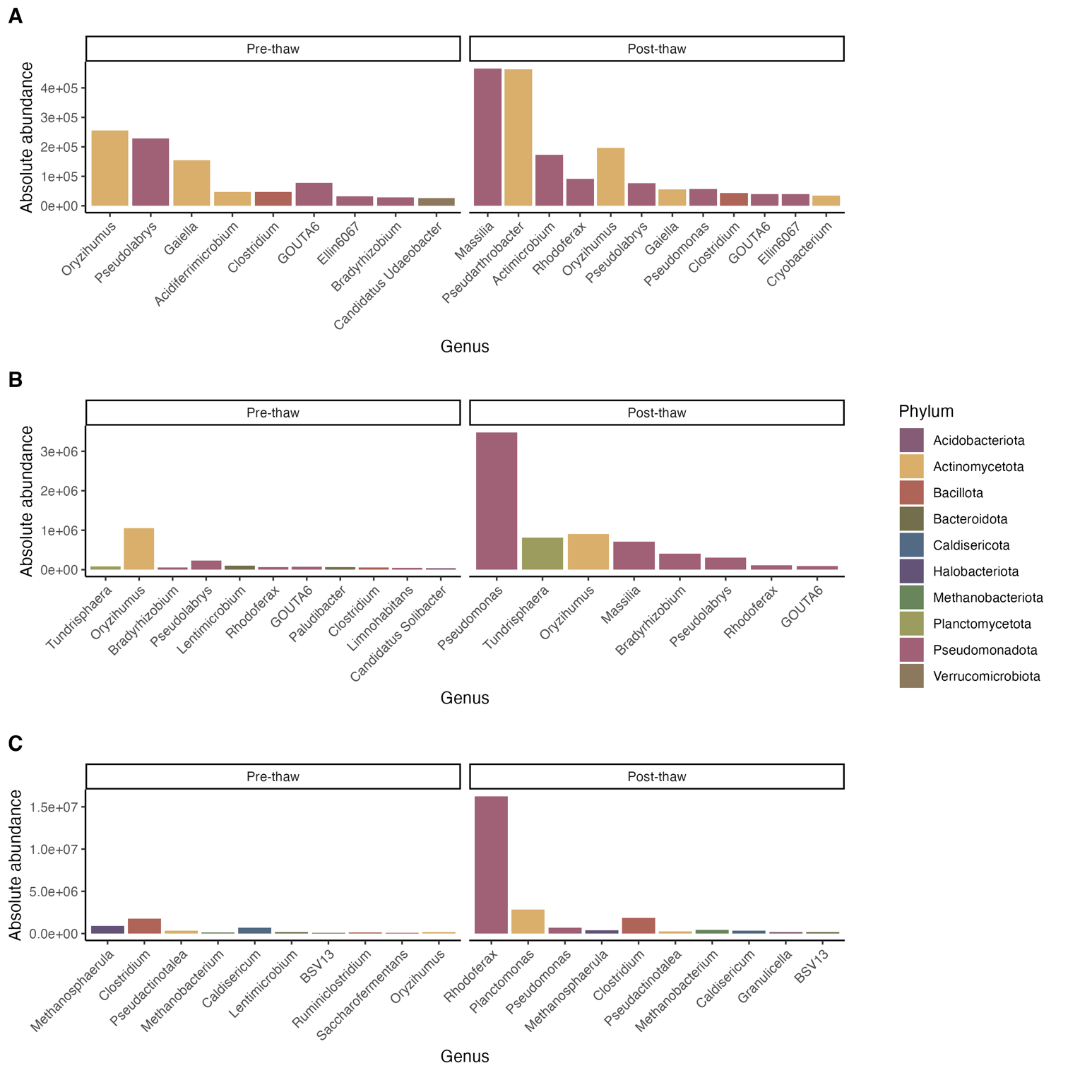
**

**Supplementary Figure 4.** Absolute abundance of top genera and their corresponding phyla in each site before and after thaw. A) Top genera detected in CRREL permafrost tunnel samples (PT) represent ~ 53% of the pre-thaw community and ~ 66% of the post-thaw community. B) Top genera detected in CRREL Farmer’s Loop samples (FL) represent ~ 67% of the pre-thaw community and ~81% of the post-thaw community. C) Top genera detected in Barrow Experimental Observatory samples (BEO) represent ~ 70% of the pre-thaw community and ~ 89% of the post-thaw community.
